# Proline metabolism regulates replicative lifespan in the yeast *Saccharomyces cerevisiae*

**DOI:** 10.15698/mic2019.10.694

**Published:** 2019-09-24

**Authors:** Yukio Mukai, Yuka Kamei, Xu Liu, Shan Jiang, Yukiko Sugimoto, Noreen Suliani binti Mat Nanyan, Daisuke Watanabe, Hiroshi Takagi

**Affiliations:** 1 Department of Frontier Bioscience, Faculty of Bioscience, Nagahama Institute of Bio-Science and Technology, 1266 Tamura, Nagahama, Shiga 526-0829, Japan.; 2 Division of Biological Science, Graduate School of Science and Technology, Nara Institute of Science and Technology, 8916-5 Takayama, Ikoma, Nara 630-0192, Japan.

**Keywords:** proline, replicative lifespan, stress response, amino acid metabolism, yeast

## Abstract

In many plants and microorganisms, intracellular proline has a protective role against various stresses, including heat-shock, oxidation and osmolarity. Environmental stresses induce cellular senescence in a variety of eukaryotes. Here we showed that intracellular proline regulates the replicative lifespan in the budding yeast *Saccharomyces cerevisiae*. Deletion of the proline oxidase gene *PUT1* and expression of the γ-glutamate kinase mutant gene *PRO1-I150T* that is less sensitive to feedback inhibition accumulated proline and extended the replicative lifespan of yeast cells. Inversely, disruption of the proline biosynthetic genes *PRO1, PRO2*, and *CAR2* decreased stationary proline level and shortened the lifespan of yeast cells. Quadruple disruption of the proline transporter genes unexpectedly did not change intracellular proline levels and replicative lifespan. Overexpression of the stress-responsive transcription activator gene *MSN2* reduced intracellular proline levels by inducing the expression of *PUT1*, resulting in a short lifespan. Thus, the intracellular proline levels at stationary phase was positively correlated with the replicative lifespan. Furthermore, multivariate analysis of amino acids in yeast mutants deficient in proline metabolism showed characteristic metabolic profiles coincident with longevity: acidic and basic amino acids and branched-chain amino acids positively contributed to the replicative lifespan. These results allude to proline metabolism having a physiological role in maintaining the lifespan of yeast cells.

## INTRODUCTION

Microorganisms and plants are exposed to various environmental stresses during their lifespan and have developed a variety of adaptation strategies against these stresses (e.g., altered membrane composition, induction of stress-responsive proteins, and accumulation of compatible solutes) [1]. Proline is known to function *in vitro* as a stress protectant, namely a protein and membrane stabilizer, protein-folding chaperone, and reactive oxygen species (ROS) scavenger [2–4]. Proline also functions as a compatible solute, a small molecule whose presence in large numbers helps organisms survive extreme osmotic stress [1, 5]. In the budding yeast *Saccharomyces cerevisiae*, the synthesis of proline from glutamate in the cytoplasm is catalyzed by three enzymes: Pro1p (γ-glutamyl kinase), Pro2p (γ-glutamyl phosphate reductase), and Pro3p (Δ^1^-pyrroline-5-carboxylate (P5C) reductase) [6, 7]. Proline is also synthesized from arginine by two enzymes: Car1p (arginase) and Car2p (ornithine transaminase) [8]. On the other hand, excess proline is degraded to glutamate in mitochondria by two enzymes, Put1p (proline oxidase) and Put2p (P5C dehydrogenase), and inhibits the enzymatic activity of Pro1p by negative feedback inhibition [9]. Unlike bacterial and plant cells, yeast cells do not elevate proline levels in response to various stresses [10]. To date, however, we have found that the Asp154Asn and Ile150Thr variants of Pro1p were less sensitive to feedback inhibition, leading to excess proline synthesis [11, 12]. Thus, *S. cerevisiae* cells accumulated proline by expressing the *PRO1-D154N* and *PRO1-I150T* genes encoding the above variants and by disrupting the *PUT1* gene encoding proline oxidase [13]. Proline accumulation was shown to confer stress tolerance to freezing, desiccation, oxidation, and ethanol on yeast cells [11, 13–19].

Numerous genes involved in determining lifespan have been identified in a variety of model organisms including yeast [20–23]. The replicative lifespan of a yeast cell is defined as the number of daughter cells produced from a mother cell before dying [24] and may be similar to the aging of mitotically active cells in multicellular organisms [25, 26]. In addition to metabolic enzymes [27–32], it has also been shown that stress response factors are implicated in the replicative lifespan: e.g., protein kinase Sch9p and transcription factors Msn2p and Msn4p [28, 33]. Sch9p is involved in the transactivation of osmostress-responsive genes and is required for TORC1-mediated regulation of ribosomal biogenesis and translation initiation through phosphorylation by Tor1p kinase [34]. Msn2p/Msn4p are transcriptional activators in response to various stress conditions [35, 36].

Since proline functions as a stress protectant in yeast, as described above, we wondered whether intracellular proline is involved in the yeast replicative lifespan. Accordingly, the replicative lifespan of yeast mutants with altered proline content was measured and the data was statistically correlated to intracellular proline and other amino acids.

## RESULTS AND DISCUSSION

### Extended replicative lifespan observed in yeast cells with increased intracellular proline content

**Figure 1:**
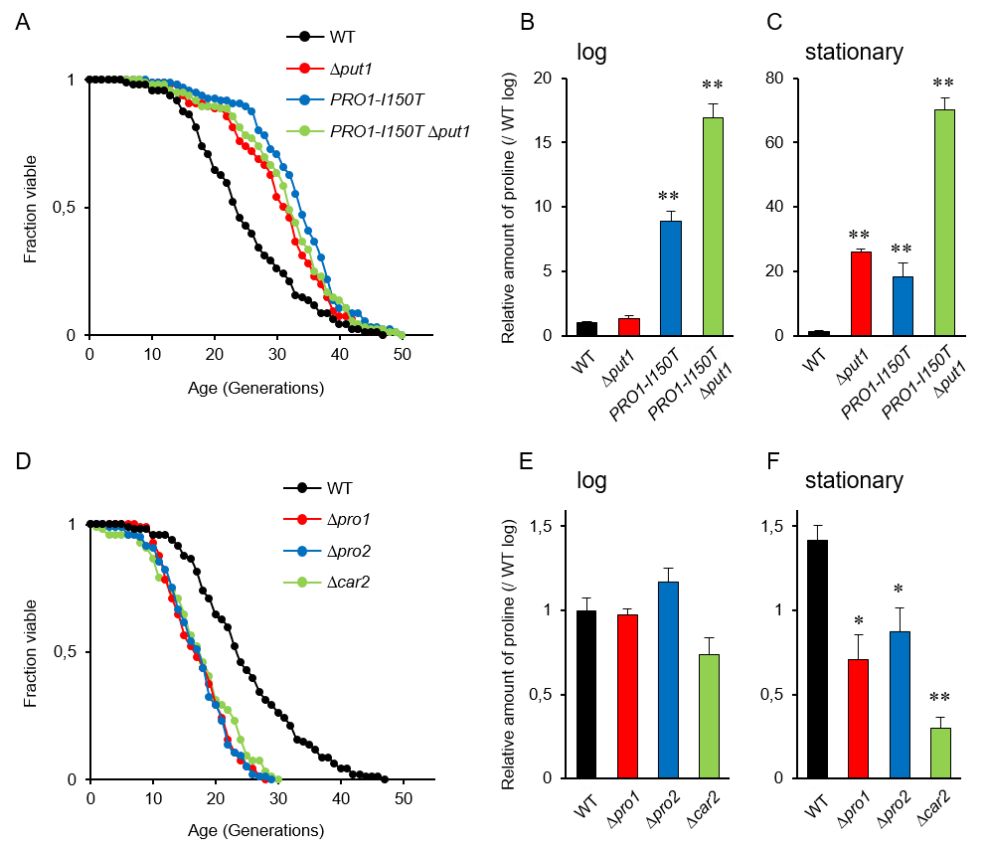
FIGURE 1: Intracellular proline regulates the yeast replicative lifespan. (A and D) Lifespan of yeast cells with increased or decreased proline levels. The data for the wild-type strain (WT; BY4741) was the same as Figure 1A and 1D. The results of other mutants, which were analyzed independently, and WT were combined. **(B, C, E and F)** Intracellular proline levels in the indicated strains at log and stationary phases compared with that in WT at log phase. The values are the means and standard deviation of results from three independent experiments. *, *p* < 0.05; **, *p* < 0.01, *versus* WT calculated with the Dunnett's test.

It is known that accumulation of proline in yeast cells confers tolerance to various stresses, e.g., freezing, desiccation, oxidation, and ethanol [9], and that several stress-resistant mutants are long lived [28]. Accordingly, we expected that proline accumulation would extend the replicative lifespan of yeast cells. The deletion mutant of *PUT1*, encoding proline oxidase, and a mutant expressing *PRO1-I150T*, which encodes the γ-glutamyl kinase variant (Ile150Thr) with desensitization to feedback inhibition by proline [12], exhibited markedly longer lifespans on YPD medium compared with the wild-type strain (WT) (**Figure 1A**; replicative lifespan measurements from this study are shown in **Table 1**). The Δ*put1 PRO1-I150T* double-mutant did not show further lifespan extension compared with each single mutant.

**TABLE 1.** Replicative lifespan of yeast mutants involved in proline metabolism.

Allele	Replicative lifespan						
	**Average**	**±**	**SD**	**Percentage change (%)**	***p* value^b^**	**Maximum**	***n***
**BY4741**							
WT	24.9	±	8.5	-	-	47	96
Δ*put1*	30.5	±	7.9	22.5	4.2×10^−6^	47	96
*PRO1-I150T*	33.3	±	7.5	33.7	4.9×10^−11^	50	96
Δ*put1 PRO1-I150T*	31.5	±	8.2	26.5	1.0×10^−7^	50	96
Δ*car2*	17.6	±	6.4	−29.3	1.1×10^−8^	30	96
Δ*pro1*	17.3	±	4.9	−30.5	5.1×10^−11^	28	96
Δ*pro2*	17.1	±	5.3	−31.3	5.9×10^−11^	29	96
*MSN2*(OE)^a^	16.3	±	4.9	−34.5	2.7×10^−13^	28	96
*MSN2*(OE)^a^ Δ*put1*	21.1	±	6.7	−15.3	3.2×10^−3^	38	96
Δ*msn2* Δ*msn4*	23.8	±	8.8	−4.4	5.6×10^−1^	43	48
**CAY29**^b^							
WT	22.6	±	7.3	-	-	43	48
Δ*put4* Δ*gap1* Δ*agp1* Δ*gnp1*	23.0	±	8.3	1.77	6.3×10^−1^	41	48

^a^ *MSN2*(OE) represents overexpression of the *MSN2* gene driven by the *TDH3* promoter.

^b^ The *p* value was calculated using a Wilcoxon rank-sum test relative to the wild-type strain (WT).

We confirmed the proline accumulation in the Δ*put1* and *PRO1-I150T* mutants. As previously reported [18], the proline level of the Δ*put1* mutant during logarithmic growth in YPD medium was comparable to that of WT, while that of the *PRO1-I150T* mutant was 9-fold higher than that of WT (**Figure 1B**). In the stationary phase (after three days of cultivation), both the Δ*put1* and *PRO1-I150T* mutant strains accumulated high levels of proline compared with WT (**Figure 1C**). The proline levels of WT cells in the stationary phase were almost the same (1.4-fold) as those in the log phase. These results suggest that an increase in proline levels extends the replicative lifespan of yeast cells when grown aerobically in YPD medium. In addition, even though the Δ*put1 PRO1*-*I150T* double-mutant lifespan was similar to that of each single mutant, the double-mutant strain highly accumulated proline in the log and stationary phases (17- and 70-fold, respectively, higher than WT in each phase). These results indicate that excess proline does not lead to further lifespan extension.

In the Δ*put1* mutant, proline is not accumulated in the log phase probably due to high consumption of proline for cell growth. In contrast, cell proliferation will stop in the stationary phase, leading to proline accumulation that could delay the replicative aging, consequently extending lifespan. This speculation might be related to our previous results that the stationary phase-induced genes were mostly up-regulated in aged cells [38].

### Shortened replicative lifespan observed in yeast cells with reduced intracellular proline content

Since the accumulation of proline extended the replicative lifespan of yeast cells, we assumed that a decrease of intracellular proline levels would shorten its lifespan. To address this assumption, we examined the effect of deleting three genes involved in proline biosynthesis on the replicative lifespan. The *PRO1* and *PRO2* genes encode the first and second enzymes, respectively, in proline biosynthesis from glutamate [6, 7]. The *CAR2* gene encodes the second enzyme in arginine degradation to supply glutamate-γ-semialdehyde as a proline precursor [8]. As expected, deletion of *PRO1, PRO2*, or *CAR2* markedly shortened the lifespan on YPD medium (**Figure 1D**). Intracellular proline levels in the Δ*pro1*, Δ*pro2*, and Δ*car2* mutants were comparable to those in WT when these cells were cultivated to the log phase (**Figure 1E**). However, when cultivated to the stationary phase, all the mutants had significantly lower level of proline compared with that of WT (**Figure 1F**). These results suggest that a decrease in the proline levels shortens the replicative lifespan of yeast cells when grown aerobically in YPD medium.

It has been reported that *S*. *cerevisiae* has four proline transporter genes: two general amino acid permease genes, *GAP1* and *AGP1*, the proline permease gene *PUT4*, and the glutamine permease *GNP1* gene [37]. Hence, we speculated that a deficiency in proline permease activity would decrease the intracellular proline levels, leading to a shortened lifespan. However, the quadruple deletion of the proline transporter genes did not change the lifespan of yeast cells on YPD medium (Figure S1A). This result might be reasonable because the proline level in the quadruple mutant seemed to be comparable to the level of WT (Figure S1B). Therefore, glutamate taken up from YPD medium might be utilized to proline synthesis in the cell even when the proline transporter genes are deficient.

### The stress-responsive transcription activator genes MSN2/MSN4 are involved in replicative lifespan

**Figure 2:**
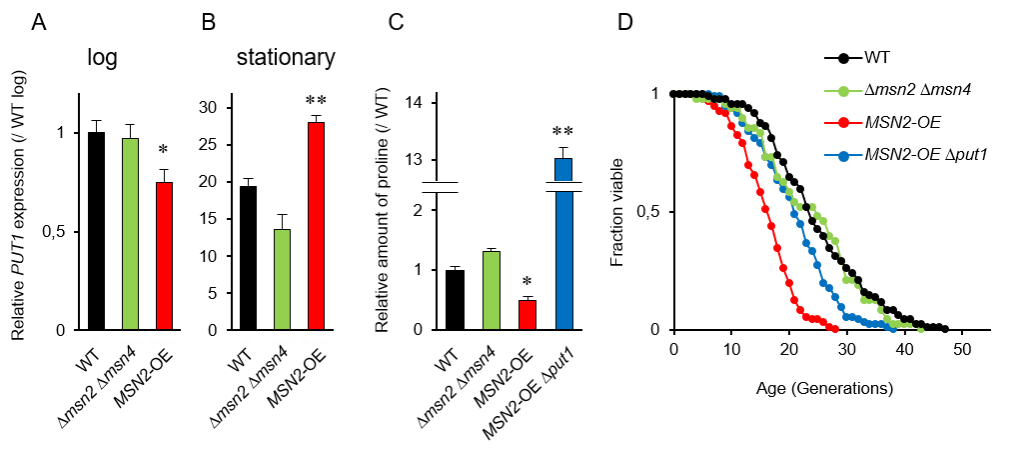
FIGURE 2: The stress-responsive transcription activator genes *MSN2/MSN4* are involved in replicative lifespan. (A and B) The expression of *PUT1* in Δ*msn2* Δ*msn4* double deletion strain and *MSN2*-overexpressing strain at log and stationary phases compared with that in the wild-type strain (WT; BY4741) at log phase. The values are the means and standard deviation of results from three independent experiments. *, *p* < 0.05; **, *p* < 0.01, *versus* WT calculated with the Student's *t*-test. **(C)** Intracellular proline levels in the indicated strains compared with WT at stationary phase. The values are the means and standard deviation of results from three independent experiments. *, *p* < 0.05; **, *p* < 0.01, *versus* WT calculated with the Dunnett's test. **(D)** Replicative lifespan of the indicated strains. The data for WT was the same as Figure 1A and 1D. The results of other mutants, which were analyzed independently, and WT were combined.

Since the promoter region in *PUT1* contains two stress response elements (STREs), which are bound by two paralogous transcriptional activators Msn2p and Msn4p [36], we postulated that *MSN2* and *MSN4* regulate the replicative lifespan by mediating the proline levels. RT-qPCR analysis showed that the induction of *PUT1* transcription in WT in the stationary phase was about 20-fold greater than that in the log phase (**Figure 2A** and **2B**). In the stationary phase, overexpression of *MSN2* driven by the *TDH3* promoter significantly enhanced the *PUT1* transcription and decreased proline level (**Figure 2B** and **2C**). On the other hand, when *MSN2* and *MSN4* were simultaneously deleted, there were no significant changes in the transcript level of *PUT1* and the proline level compared to those of WT. Thus, the Δ*msn2* Δ*msn4* double-mutant exhibited a normal replicative lifespan (**Figure 2D**). Overexpression of *MSN2* remarkably shortened the replicative lifespan, which was restored almost to the lifespan of WT by deletion of *PUT1*, although the proline level in the *MSN2*-overexpressing and *put1*-deleted strain was 13-fold higher than that in WT (**Figure 2C** and **2D**). These results support our hypothesis that intracellular proline, in part, modulates the replicative lifespan.

To verify the above results that intracellular proline levels, which significantly change in the stationary phase, might regulate longevity, we performed correlation analysis between the proline levels in the stationary phase and the mean lifespan. The correlation coefficient (R=0.687) suggests that intracellular proline positively regulates longevity of yeast cells (Figure S2).

The transcript level of *PUT1*, when driven by the constitutive *TDH3* promoter, was not enhanced in the log phase. This result suggests that the stability of the *PUT1* transcripts depends on the growth phase. We previously observed that many genes that are induced during the stationary phase are transactivated at the early stages of replicative senescence of yeast cells [38]. Similarly, the mRNA levels of *PUT1* were also increased in 11^th^ generation cells compared with 1^st^ generation cells [38]. Therefore, the age-dependent expression of *PUT1* suggests that the control of intracellular proline levels is important for yeast cells during senescence. The stress-responsive transcription activator Msn2p, potentially also Msn4p, might be activated at the early senescence stage to upregulate the senescence-specific genes including *PUT1*, resulting in maintaining the lifespan of yeast cells.

### Amino acid analysis of yeast mutants involved in proline metabolism

To determine the metabolic effects of increasing and decreasing intracellular proline levels, we analyzed the amino acid contents in the yeast strains used in this study in the log and stationary phases (Figure S3). In WT, half of the amino acids were decreased in the stationary phase relative to the log phase, whereas leucine and valine contents were increased. Thus, the changes took place in the stationary phase, but proline was unique in showing a correlation to the lifespan.

**Figure 3:**
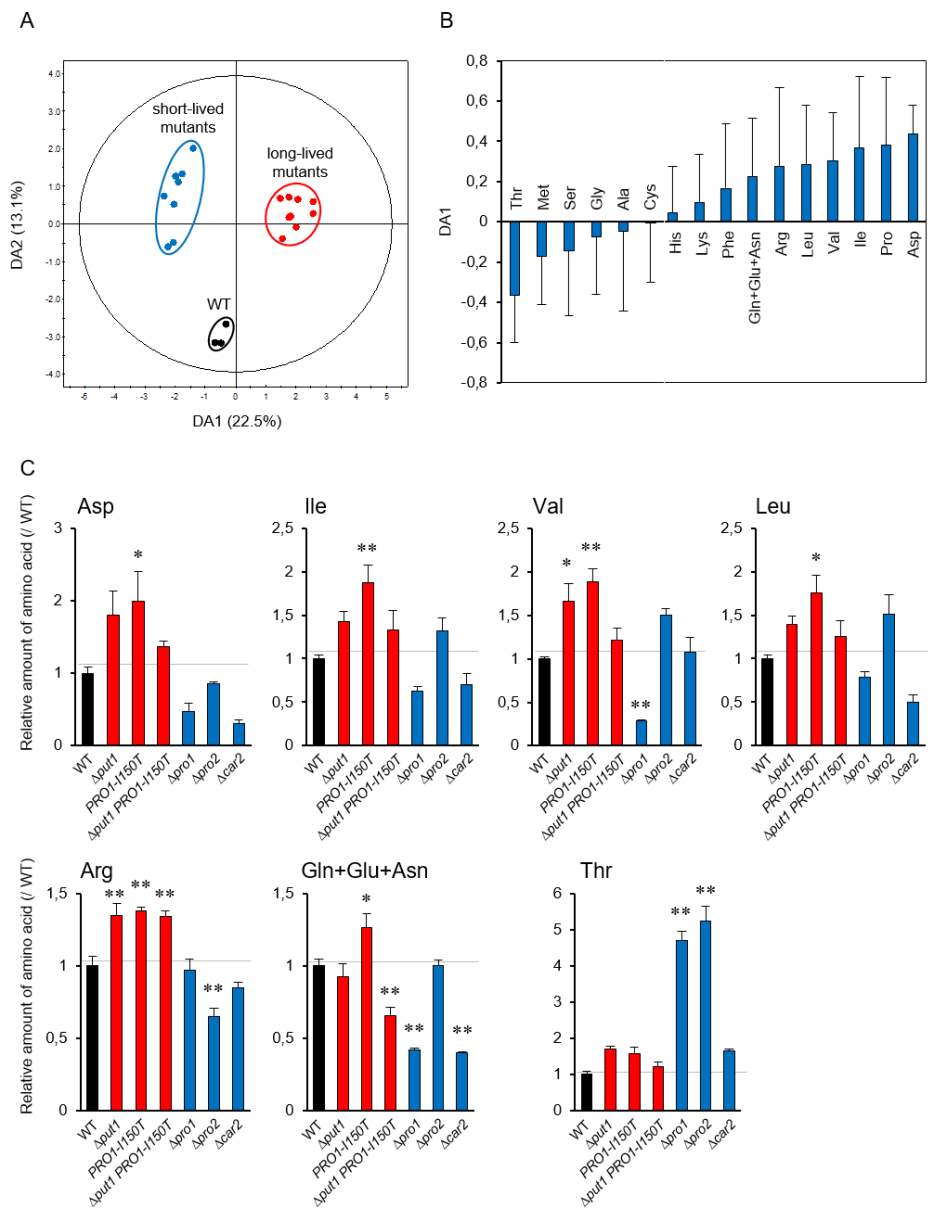
FIGURE 3: OPLS-DA of amino acid analysis data from yeast mutants involved in proline metabolism. **(A)** Score plot of OPLS-DA for the wild-type strain (WT; BY4741) and short- and long-lived mutants involved in proline metabolism. **(B)** Loading plot for DA1 in panel (A). **(C)** Each point represents an individual batch from WT and the mutants for indicated gene at log phase. The values are the means and standard deviation of results from three independent experiments. *, *p* < 0.05; **, *p* < 0.01, *versus* WT calculated with the Dunnett's test.

To link metabolism of proline to individual amino acids [38], we performed a principal component analysis (PCA) using the amino acid contents in the yeast mutants involved in proline metabolism to investigate the lifespan-dependent amino acid metabolism. The PCA score plot did not indicate the expected separation of these strains in the log and stationary phases (Figure S4A and S4B). Accordingly, to identify which amino acids contribute to the lifespan determination, we performed orthogonal projection to latent structure discriminant analysis (OPLS-DA) using the above amino acid dataset. The OPLS-DA score plot indicates a clear separation between WT and the short- and long-lived mutants (**Figure 3A**). This separation depended on lifespan along the DA1 axis. The loading plot for DA1 showed that, in addition to proline, acidic amino acids (aspartate and glutamate), branched-chain amino acids (isoleucine, valine, and leucine), and basic amino acids (arginine and lysine) positively contributed to longevity, whereas serine, threonine, and methionine were negatively involved (**Figure 3B**). These results were confirmed by the individual results (**Figure 3C**). Aspartate, isoleucine, valine, leucine, and arginine tended to be increased in the proline-increased mutants and decreased in the proline-decreased mutants. Threonine was remarkably increased in the proline-decreased mutants. These results suggest that the metabolic changes due to changing intracellular amino acid levels cause the lifespan determination.

In this study, we examined the phenotypes of various strains derived from BY4741 and CAY29 with S288C genetic background. It should be noted that genotype-phenotype outcomes are not always the same in all strains because of the influence of nuclear genome variation and that of the mitochondrial genome. To demonstrate our hypothesis that proline metabolism has a physiological role of influencing the replicative aging of yeast, it is necessary to construct other strains with different genetic background and to check their phenotypes. We initially focused on the involvement of proline in the ‘replicative' aging of yeast cells. From our results, it is intriguing whether proline accumulated at the stationary phase extends ‘chronological' lifespan of yeast cells.

Recently, higher proline levels were found to increase longevity in *Caenorhabditis elegans* [39]. Another study also showed that impaired insulin/IGF-1 signaling extends the *C. elegans* lifespan by promoting mitochondrial proline catabolism, while proline supplementation extends the lifespan [40]. These results suggest that proline directly affects longevity in *C. elegans*, but change in amino acid metabolism caused by the intracellular proline might have some effect on longevity in yeast. However, little is known about the roles of proline regulating the replicative lifespan of yeast cell. We previously found that deletion of genes involved in converting glutamate to γ-aminobutyric acid could increase the replicative lifespan [31] and lead to increased conversion of glutamate to α-ketoglutarate and other TCA cycle intermediates that maintain mitochondrial respiratory function. The replicative lifespan is also regulated by genes encoding metabolic enzymes, the hexokinase isoenzyme 2 Hxk2p, the alcohol dehydrogenase Adh1p, and the saccharopine dehydrogenase Lys9p [27–29]. Yeast is a unique and powerful model for investigation of the effects of these genes on lifespan in eukaryotic cells. Furthermore, breeding of industrial yeast strains with accumulation of proline, which is correlated with lifespan extension, could contribute to an improvement in fermentation ability and compound productivity.

## MATERIALS AND METHODS

### Strains and media

The *S. cerevisiae* strains used in this study were derived from BY4741 or CAY29, which are S288C-derivative laboratory strains, and are listed in Table S1. The deletion strains were obtained from the Yeast MATa Collection (Open Biosystems, AL) for BY1-put1, BY1-car2, BY1-pro1, and BY1-pro2 and P. O. Ljungdahl for CAY191 [37]. The other mutant and overexpression strains (BY1-PRO1-I150T, BY1-put1-PRO1-I150T, BY1-MSN2OE, BY1-MSN2OE-put1, and BY1-msn2/4) were constructed in this study (Table S1). Gene manipulations such as gene overexpression, and gene deletions were carried out through genomic integration as described previously [41]. Oligonucleotide primers used in this study are listed in Table S2. We first integrated a mutated *PRO1* gene by homologous recombination by the *URA3* marker. In order to construct *PRO1-I150T* mutant strains, a linearized plasmid, pRS406-I150TPRO1 [42], which expresses the Ile150Thr variant of Pro1p, was introduced into strain BY4741 or BY1-put1. After the Ura^+^ transformants grown on SC-uracil medium (20 g/L glucose, 6.7 g/L Bacto yeast nitrogen base without amino acids [Difco Laboratories, Detroit, MI], 2 g/L drop-out mixture) were cultured in YPD medium (20 g/L glucose, 10 g/L Bacto yeast extract, 20 g/L Bacto peptone) at 30°C for 24 h with shaking, diluted to the same media, incubated for several days, strains BY1-PRO1-I150T and BY1-put1-PRO1-I150T that have excised the plasmid and lost one of the two copies of the duplicated region by homologous crossover were obtained from 5-fluoroorotic acid-containing plates [43].

The *MSN2* overexpression cassette consisting of the *URA3* marker (containing both native promoter and terminator of the *URA3* gene) and a constitutive, a strong promoter of the *TDH3* gene (P_*TDH3*_) was amplified via polymerase chain reaction (PCR) using a set of primers (MSN2_up_F and MSN2_from_1_to_534_R). This cassette was then integrated between the native promoter of *MSN2* gene (P_*MSN2*_) and the open reading frame of *MSN2* gene through yeast transformation. Following that, positive clones were confirmed using another set of primers (TDH3_up_F(-132_-108) and MSN2_R2). For *MSN2* deletion, this pair of primers was used (MSN2+URA3-Fw and MSN2+URA3-Rv) to amplify the disruption cassette. For gene deletions, plasmid pUG6 harboring geneticin-resistant gene was used as a template to amplify the deletion cassettes using specific sets of primers (Fw Δmsn4-kanMX and Rv Δmsn4-kanMX). YPD medium was used for routine cultures, and G418 (200 µg/mL) was added to YPD medium when required.

### Replicative lifespan determination

The replicative lifespan was assayed as previously described [38]. A sample of yeast cells was spread onto YPD agar plates containing 10 μg/mL phloxine B. Virgin daughter cells were selected and subjected as sample cells to lifespan analysis. Daughter cells were removed with a dissecting needle and scored every 2 h. For each of at least 48 cell lines, buds from each mother cell were counted until division of living cells ceased on YPD agar plates or cells were stained with phloxine B. **Table 1** shows all of the replicative lifespan data. For statistical analysis, lifespan data sets were compared by a Wilcoxon rank-sum test. Two strains were stated to have a significant difference in lifespan when *p* < 0.05.

### Reverse transcription-quantitative polymerase chain reaction (RT-qPCR)

The isolation of total RNA from yeast cells and subsequent RT-qPCR analysis were carried out as previously described [38]. Total RNA was extracted from yeast cells using a RNeasy Mini Kit (Qiagen, Valencia, CA, USA). Extracted total RNAs were subjected to cDNA synthesis using a PrimeScript RT Master Mix (Takara Bio, Shiga, Japan) with random primers. qPCR was carried out using a SYBR Premix Ex TaqII (Takara Bio) on a Thermal Cycler Dice Real Time System II (Takara Bio). The relative mRNA concentration was normalized to that of the *RDN18* gene transcript as an internal control. For statistical analysis, RT-qPCR data sets were compared by a Student's *t*-test. Two strains were stated to have a significant difference in lifespan when *p* < 0.05.

### Amino acid quantification

For the determination of intracellular proline and other amino acids, yeast cells were grown in 5 mL YPD medium at 30°C to the log and stationary phases for 8 h and 24 h, respectively, with shaking (120 rpm); 500 μL cell suspension were removed, the cells were washed twice with 0.9% NaCl and suspended in 0.5 mL distilled water. The 1.5 mL microcentrifuge tube containing cells was transferred to a boiling water bath, and intracellular amino acids were extracted by boiling for 20 min. After centrifugation (15,000 x *g*, 5 min, 4°C), each supernatant was analyzed quantitatively using an amino acid analyzer (JLC500/V, Jeol, Tokyo, Japan). Amino acid contents were calculated as a percentage of dry weight. For statistical analysis, quantification data sets were compared by a Dunnett's test. Two strains (each of mutants vs. the wild-type strain) were stated to have a significant difference in amino acid contents when *p* < 0.05.

### Multivariate Analysis

The data sets from the amino acid analysis were judged in all cases by principal component analysis (PCA) and orthogonal projection to latent structure (OPLS) using SIMCA-P+ 12.0.1 (Umetrics, Sweden).

## SUPPLEMENTAL MATERIAL

Click here for supplemental data file.

All supplemental data for this article are available online at http://www.microbialcell.com/researcharticles/2019a-mukai-microbial-cell/.

## Abbreviations:

OPLS-DA: – orthogonal projection to latent structure discriminant analysis,
PCA: – principal component analysis,
WT: – wild type.
