# Supplementary material for: Proline metabolism regulates replicative lifespan in the yeast Saccharomyces cerevisiae
Source: Microb Cell. 2019 Sep 24;6(10):482–90. doi: 10.15698/mic2019.10.694 (PMC6780008; doi:10.15698/mic2019.10.694)
Supplement: Supplementary file 1 [file mic-06-482-s01.pdf]

Supplemental Data

Proline metabolism regulates replicative lifespan in the yeast *Saccharomyces cerevisiae*

Yukio Mukai[1, *], Yuka Kamei[1], Xu Liu[1], Shan Jiang[1], Yukiko Sugimoto[2], Noreen Suliani binti Mat Nanyan[2], Daisuke Watanabe[2], Hiroshi Takagi[2, *]

[1]Department of Frontier Bioscience, Faculty of Bioscience, Nagahama Institute of Bio-Science and Technology, 1266 Tamura, Nagahama, Shiga 526-0829, Japan

[2]Division of Biological Science, Graduate School of Science and Technology, Nara Institute of Science and Technology, 8916-5 Takayama, Ikoma, Nara 630-0192, Japan

***Corresponding Authors:** Yukio Mukai, Department of Bioscience, Faculty of Bioscience, Nagahama Institute of Bio-Science and Technology, 1266 Tamura, Nagahama, Shiga 526-0829, Japan; Tel.: +81-749-64-8163; Fax: +81-749-64-8140; E-mail: y_mukai@nagahama-i-bio.ac.jp. Hiroshi Takagi, Division of Biological Science, Graduate School of Science and Technology, Nara Institute of Science and Technology, 8916-5 Takayama, Ikoma, Nara 630-0192, Japan; Tel.: +81-743-72-5420; Fax: +81-743-72-5429; E-mail: hiro@bs.naist.jp.

This PDF file includes:

Supplementary Tables S1 - S2

Supplementary Figures S1 - S4

TABLE S1. Yeast *S. cerevisiae* strains used in the study.

Strain	Genotype	Source
BY4741 (WT)	*MAT***a** *ura3Δ0 leu2Δ0 his3Δ1 met15Δ0*	EUROSCARF
BY1-put1 (Δ*put1*)	BY4741 *put1*::*kanMX4*	Yeast MATa Collection
BY1-PRO1-I150T (*PRO1-I150T*)	BY4741 *PRO1-I150T*	This study
BY1-put1-PRO1-I150T (Δ*put1 PRO1-I150T*)	BY4741 *put1*::*kanMX4 PRO1-I150T*	This study
BY1-car2 (Δ*car2*)	BY4741 *car2*::*kanMX4*	Yeast MATa Collection
BY1-pro1 (Δ*pro1*)	BY4741 *pro1*::*kanMX4*	Yeast MATa Collection
BY1-pro2 (Δ*pro2*)	BY4741 *pro2*::*kanMX4*	Yeast MATa Collection
BY4741u (WT)	*MAT***a** *ura3Δ0*	[44]
BY1-MSN2OE (MSN2-OE)	BY4741u P_{MSN2}*::URA3-*P_{TDH3}	This study
BY1-MSN2OE-put1 (MSN2-OE Δ*put1*)	BY4741u P_{MSN2}*::URA3-*P_{TDH3} *put1Δ::kanMX*	This study
BY1-msn2/4 (Δ*msn2* Δ*msn4*)	BY4741u *msn2::URA3 msn4::kanMX6*	This study
CAY29 (WT)	*MAT***a** *ura3-52*	[45]
CAY191 (Δ*put4* Δ*gap1* Δ*agp1* Δ*gnp1*)	*MAT***a** *ura3-52 put4Δ gap1Δ agp1Δ gnp1Δ*	[37]

TABLE S2. Oligonucleotide primers used in the study.

Oligonucleotide	Sequence (5’ → 3’)
MSN2_up_F	TTGTTTCCAGCGAAAGAGAC
MSN2_from_1_to_534_R	TGAAGTTTGAGGCGATAAATTAGT
TDH3_up_F(-132_-108)	ACGGTAGGTATTGATTGTAATTCTG
MSN2_R2	ATCAAAGGCACAGCAGACT
MSN2+URA3-Fw	GTATCTTCCTCATATTTTTCGGGAAGATCACAACAGTAGTAGCAAGGTATTTCATACGCCAAGAGGCTACGATTCGGTAATCTCCGAG
MSN2+URA3-Rv	AACAATAAGCCGTAAGCTTCATAAGTCATTGAACAGAATTATCTTATGAAGAAAGATCTATCGAATTAGTAATAACTGATATAATTAAATTG
Fw Δmsn4-kanMX	TTCGGCTTTTTTTTCTTTTCTTCTTATTAAAAACAATATAATGGGTAAGGAAAAGACTCA
Rv Δmsn4-kanMX	TAGCTTGTCTTGCTTTTATTTGCTTTTGACCTTATTTTTTTAGAAAAACTCATCGAGCA

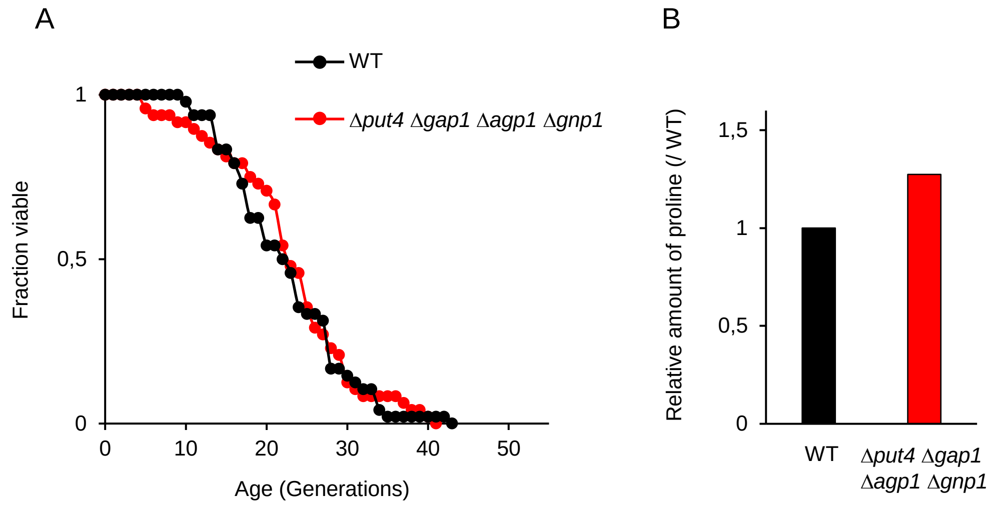

FIGURE S1. Replicative lifespan **(A)** and intracellular proline levels **(B)** in the wild-type (WT; CAY29) and quadruple deletion mutant (CAY191) strains of proline transporter genes. The experiment **(B)** was done on YPD medium with only one replica.

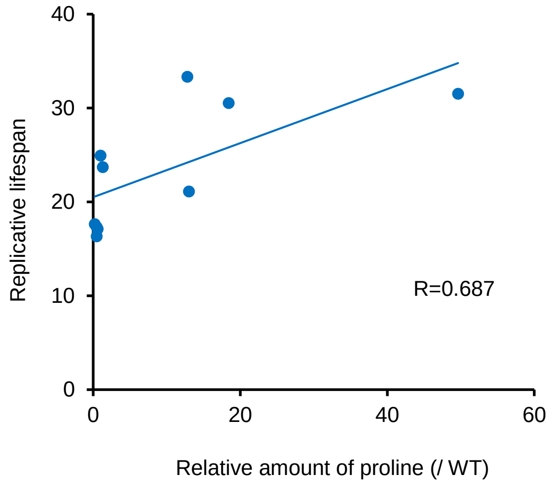

FIGURE S2. Correlation analysis between proline levels and replicative lifespan. All strains shown in Figures 1 and 2 were used in this analysis. Scatter plot of proline levels and lifespan in the wild-type (WT; BY4741) and mutant strains at stationary phase.

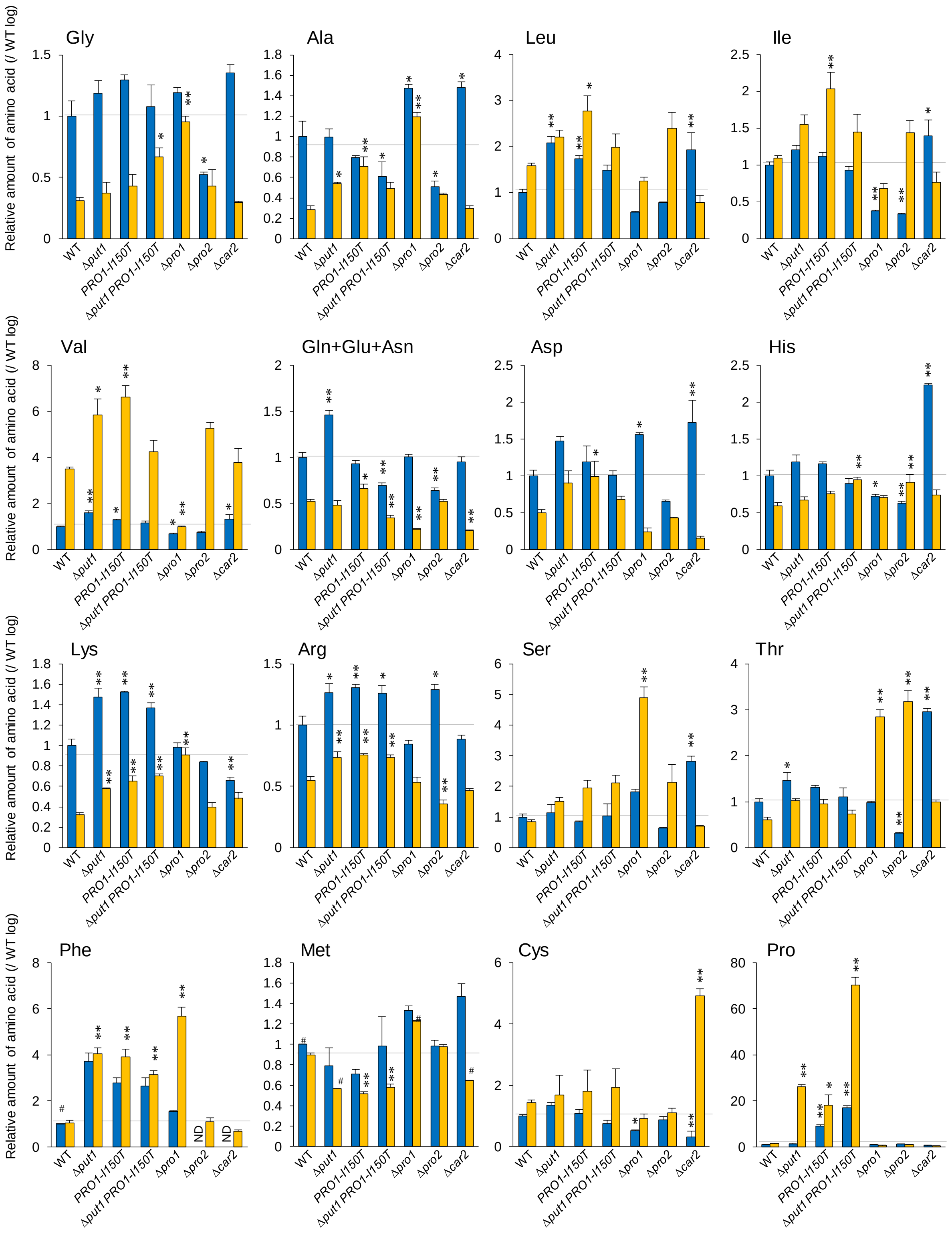

FIGURE S3. Amino acid analysis of yeast mutants involved in proline metabolism at log phase (blue) and stationary phase (orange). The values are the means and standard deviation of results from three independent experiments. *, $p < 0.05$; **, $p < 0.01$, *versus* the wild-type strain (WT; BY4741) calculated with the Dunnett's test. #, detected in only one sample; ND, not detected.

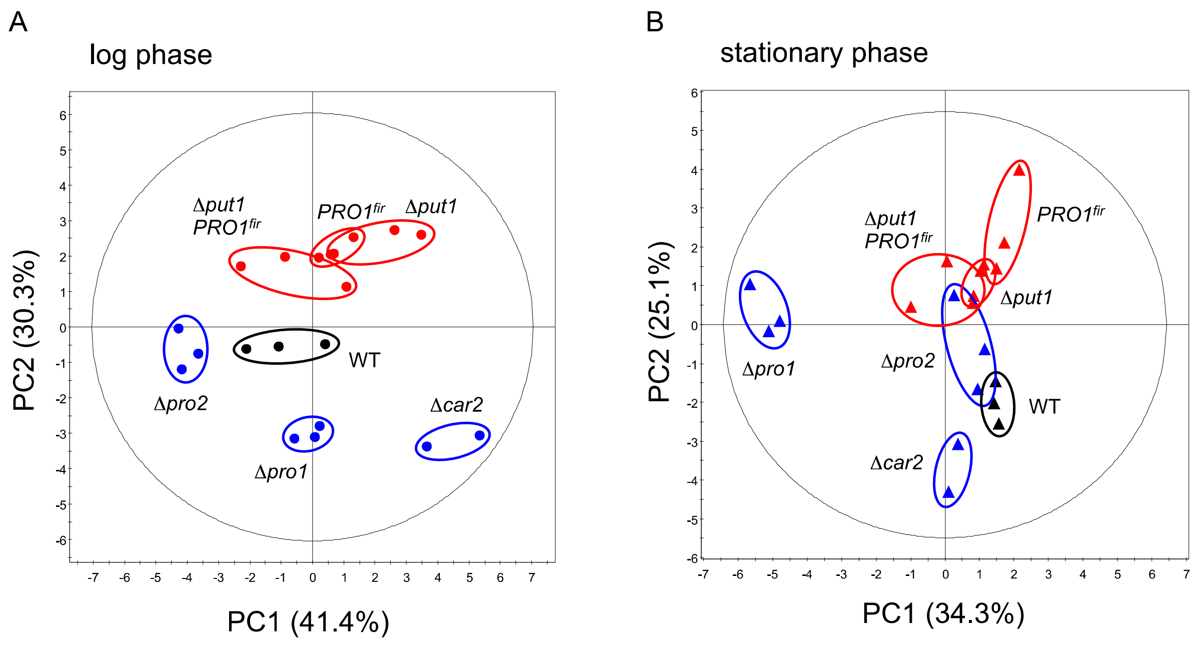

FIGURE S4. PCA score plot of amino acid analysis data from yeast mutants involved in proline metabolism. Each point represents an individual batch from the wild-type strain (WT; BY4741) and the mutants for indicated gene in the log phase (A) and the stationary phase (B). Note that *PRO1*^{fir} indicates *PRO1-I150T* described in the text.
